# Self-interest, positional concerns and distributional considerations in healthcare preferences

**DOI:** 10.1007/s10198-023-01597-4

**Published:** 2023-05-22

**Authors:** Aemiro Melkamu Daniel, Job van Exel, Caspar G. Chorus

**Affiliations:** 1 Department of Economics, Swedish University of Agricultural Sciences, Ulls Väg 27, 756 51 Uppsala, Sweden; 2 Erasmus School of Health Policy & Management, Erasmus University Rotterdam, P.O. Box 1738, 3000 DR Rotterdam, The Netherlands; 3 Erasmus Centre for Health Economics Rotterdam (EsCHER), Erasmus University Rotterdam, P.O. Box 1738, 3000 DR Rotterdam, The Netherlands; 4 Erasmus Choice Modelling Centre (ECMC), Erasmus University Rotterdam, P.O. Box 1738, 3000 DR Rotterdam, The Netherlands; 5 Faculty of Industrial Design Engineering, Delft University of Technology, Landbergstraat 15, 2628 CE Delft, The Netherlands; 6 Faculty of Technology, Policy and Management, Delft University of Technology, Jaffalaan 5, 2628BX Delft, The Netherlands

**Keywords:** Decision making, Distributional considerations, Healthcare, Positional concerns, Self-interest, Waiting times

## Abstract

Efficiently allocating scarce healthcare resources requires nuanced understanding of individual and collective interests as well as relative concerns, which may overlap or conflict. This paper is the first to empirically investigate whether and to what extent self-interest (SI), positional concerns (PC) and distributional considerations (DC) simultaneously explain individual decision making related to access to healthcare services. Our investigation is based on a stated choice experiment conducted in two countries with different healthcare systems, the United States (US) and the United Kingdom (UK). The choice experiment is on allocation of medical treatment waiting times for a hypothetical disease. We carry out the investigation under two different perspectives: (i) in a socially inclusive personal perspective decision makers were asked to choose between waiting time distributions for themselves and (ii) in a social perspective decision makers were asked to make similar choices for a close relative or friend of opposite gender. The results obtained by estimating a variety of advanced choice models indicate that DC, SI and PC, in this order of importance, are significant drivers of choice behaviour in our empirical context. These findings are consistent regardless of the choice perspective and the country where decision makers live. Comparing the results from different choice perspectives, we find that US respondents who chose for their close relative or friend attach significantly larger weight to their close relative’s or friend’s waiting times as well as to the overall distribution of waiting times than US respondents who chose for themselves. Looking at differences between countries, our results show that UK respondents who made choices for themselves placed significantly larger weight on SI and DC than US respondents, while US respondents, in turn, displayed relatively stronger but not significantly different positional concerns than UK respondents. In addition, we observe that UK respondents who chose for their close relative or friend put a larger weight on DC than their US counterparts. We conclude that the methodological (data collection and analysis) approach allows for disentangling the relative importance of the three motivations and discusses the potential implications of these findings for healthcare decision making.

## Introduction

Design and implementation of effective health policies require proper understanding of public preferences involving individual and collective interests as well as relative concerns. Knowledge about such often competing and overlapping interests is imperative to leverage scarce healthcare resources. Consider, for example, a decision about the allocation of treatment waiting times for a particular medical condition subject to healthcare capacity constraints. Such a decision could be informed by analysis assuming that the decision of every individual (patient) is driven solely by rational self-interest, i.e. the individual’s own waiting time. Despite its popular use to understand patients’ preferences in several contexts, two strands of the literature on individual decision-making behaviour challenge this conventional assumption of homo economicus.

One strand of the literature relates to the idea that some people have concerns over the distribution of resources across (relevant sections of) society. This literature explains the relative importance of fairness considerations [1–3] as well as inequality aversion and maximin preferences [4]. For example, in relation to waiting time allocation, some individuals’ preferences concerning waiting time allocation policies can be guided by the shape of the distribution of waiting times across people. Another body of literature emphasizes people’s relative (or positional) concerns [5–9]. The central idea here is that people derive satisfaction (experience disutility) merely from having more (less) resources than relevant others. With reference to the example of waiting time allocation, positional concerns imply that just having a shorter (longer) waiting time than others would result in utility (disutility). The difference between positional concerns and distributional considerations is that the latter is independent of the decision maker’s own position in the distribution, i.e. the decision maker’s choice is made from behind a metaphoric ‘veil of ignorance’ [10].

This study, to our knowledge, is the first to empirically explore the relative importance of self-interest (SI), positional concerns (PC) and distributional considerations (DC) in individual decision making related to access to healthcare services. Needless to say, rational SI is a central assumption in most models of (neo)classical microeconomic theory and application, including discrete choice experiments (DCEs). The literature on PC, which traces back to Veblen [11], is growing, with notable applications related to income [8, 12, 13]; income, leisure, cars and car safety [6]; income and consumption [5], various private and public goods and bads [9, 14], as well as a theory in connection with accessibility and mobility [15]. However, the extant literature on PC lacks applications in the health domain, Solnick and Hemenway [9] and Wouters et al. [16] being exceptions. Solnick and Hemenway [9] found limited PC, while Wouters et al. [16] found no evidence of PC for health, which implies that absolute improvements in universal healthcare services always improve subjective well-being, regardless of how the improvements are distributed. However, these studies are exploratory, their designs do not control for distributional issues, and they use data from an unrepresentative and small sample. Therefore, as the authors acknowledge, their findings cannot be taken at face value. Some of the stronger evidence for PC originates from other domains, documented by studies cited above. However, these studies are essentially based on a similar “two states of the world”^1^ design, and therefore are contested on the ground that part of the reported PC can be attributed to egalitarian preferences such as minimizing inequalities (or maximizing equality). Celse [17] and El Harbi et al. [18], for example, provide experimental evidence that supports the idea that accommodating DC, which received considerable weight in these studies, results in less prominent but non-negligible PC in preferences for payoff distributions.

We contribute to the literature on individual decision making in general and in the healthcare context in particular in three ways. First, using a variety of advanced choice models, we provide a rigorous empirical investigation into the relative importance of SI, PC and DC in decision making related to waiting times for medical treatment. In connection with PC, we examine potential asymmetry between PC due to disutility from disadvantageous inequality (i.e. having a longer waiting time relative to others) and PC due to utility from advantageous inequality (i.e. having shorter waiting time of equal magnitude relative to others). In other words, we test for loss aversion behaviour, a fundamental result from “prospect theory” [19], which implies losses loom larger than equivalent gains. In addition, we identify several distributional principles, namely expected value, minimax principle, Shannon's entropy and the generalized entropy (GE) inequality index to capture various types of DC with an appropriate metric. Furthermore, we distinguish between decision makers’ distributional preferences based on choices made behind a “veil of ignorance” and those that result from choices made when decision makers know their position in the distribution of waiting times.

Our second contribution relates to the comparison we make concerning the relative importance of SI, PC and DC in preferences for waiting time allocations between decision makers choosing for themselves and decision makers choosing for a loved one. We create contexts where decision makers adopt socially inclusive personal and social perspectives when making their choices [20]. More specifically, we elicit socially inclusive personal preferences from decision makers who were asked to choose for themselves and social preferences from decision makers who were asked to choose for a close relative or friend of opposite gender. By comparing the weights decision makers attach to the different motivations when they choose for themselves and when they choose for a close relative or friend, we provide valuable insights for framing of interventions.

The third contribution lies in carrying out our empirical investigations in two countries with different healthcare systems, namely the US and the UK. Hofstede [21] argued that national insurance systems tend to reflect societal values such as solidarity, independence and predictability. Hence, if the values underlying the largely privately funded health system in the US differ from those underlying the largely publicly funded healthcare system in the UK, we may expect to observe different relative weights for SI, PC and DC. Therefore, to establish the robustness of our findings to differences in the decision-making context, we compare the relative strengths of SI, PC and DC in preferences for waiting time allocations between citizens of the US and the UK.

The empirical investigation in this study is based on a tailor-made stated choice experiment. Stated discrete choice experiments have been previously used to produce evidence on SI and PC in different ways. The most common way, implemented in [9, 14, 16], is the approach followed by Solnick and Hemenway [7], which involves a one-off choice between two states of the world, a positional state in which subjects stand in a better position relative to society average and an absolute state where subjects stand better than the positional state but worse than society average. Choice experiments involving several choices between similar, two states of the world, designs have also been used by varying only the subject’s position in the positional state keeping the absolute state unchanged [5, 6, 12]. However, Celse [17] challenged empirical evidence on PC based on such two states of the world designs by incorporating an additional state that represent DC. More specifically, Celse [17] presented subjects with a one-off choice between an absolute state, a positional state and a state where subject’s position is identical with society average and their position in the positional state. His result shows subjects attach the greatest weight to the state representing DC than PC and SI which was also found to be the case in El Harbi et al. [18] based on stated/hypothetical as well as revealed/incentive compatible preferences.

Our experiment (explained in Sect. "Choice experiment design and data") was designed in such a way that the three types of motivations could be disentangled and identified in an econometrically efficient manner, from repeated choices made between healthcare policies resulting in different distributions of waiting times across society, and different positions of a decision maker (or a close relative) on those distributions. In each country, roughly half of the respondents made choices for themselves, while the other half made choices for a close relative or friend. Our analysis involves estimation of a series of discrete choice models on data from each sub-sample.

We find that the methodological (data collection and analysis) approach to identify the relative importance of SI, PC and DC works and that these three motivations are all significant in explaining respondents’ choices for waiting time allocation in both countries, the weight for DC being larger than the weights for SI and PC. Our results indicate no evidence of loss aversion. Of all the considered distributional principles to represent DC, expected value appears to be the most suitable metric. We did not find significant differences in relative importance of the three motivations between choices made for loved ones and choices for oneself in the UK. However, US respondents who chose for a close relative or friend attached significantly larger weight to the (absolute) levels as well as to distributions of waiting times (i.e. DC) than respondents who chose for themselves. Moreover, among UK and US respondents who made choices for themselves, we find that UK respondents attached significantly larger weight to SI and DC than US respondents, while US respondents displayed relatively stronger positional concerns than UK respondents. Also, UK respondents who chose for a close relative or friend gave a larger weight to DC than US respondents. We discuss the relevance of these findings for healthcare decision making.

The remainder of this paper is structured as follows. Sect. "Choice experiment design and data" provides a thorough description of the choice experiment scenarios, design and data collection procedures. In Sect. "Modelling approach", we explain the analytical framework. The detailed results of the study are given in Sect. "Empirical results". Finally, we discuss the main findings and conclude in Sect. "Conclusions and discussion".

## Choice experiment design and data

### Choice experiment scenario

The empirical investigation in this study is based on a stated choice experiment on allocation of waiting times for medical treatment to a hypothetical disease. Participants for the choice experiment consisted of two groups. The first group (referred to hereafter as “Group one”) included people between 30 and 50 years old who have a paid job. The second group (referred to hereafter as “Group two”) was composed of people who were at least 18 years old and who had a close relative or friend of the opposite gender between 30 and 50 years old and who had a paid job.

Right before receiving the information about the hypothetical disease, participants from Group one were presented with the EuroQol EQ-5D-5L descriptive instrument^2^ [22] to assess their current state of health, whereas Group two assessed their close relative’s or friend’s health. Subsequently, participants from Group one were told to imagine that there is a disease which affects people who are between 30 and 50 years old such as themselves. Similarly, participants from Group two were informed to imagine a disease that affects people who are of the opposite gender to themselves, who are 30–50 years old and who have a paid job. In addition, participants from Group one (Group two) were told that, if affected by the disease, their (close relative’s or friend’s).

- mobility would deteriorate by two levels if their (close relative’s or friend’s) reported health state on this dimension was moderate or higher, with no change on this dimension otherwise,
- level of self-care would not change,
- ability to do usual activities would deteriorate by two levels if their (close relative’s or friend’s) reported health state on this dimension was moderate or higher, with no change on this dimension otherwise,
- pain or discomfort would deteriorate by one level if their (close relative’s or friend’s) reported health state on this dimension was severe or higher, no change on this dimension otherwise, and
- anxiety or depression would deteriorate by one level if their (close relative’s or friend’s) reported health state on this dimension was severe or higher, with no change on this dimension otherwise.

The health deterioration if affected by the disease was defined relative to the participant’s (close relative’s or friend’s) actual health state as reported by the participant in the questionnaire. It was assumed that this makes the description of deteriorations in health more realistic and imaginable to respondents.

Participants in both groups were told that this deterioration in health would persist until the person received the treatment. Hence, it was made clear to participants that if affected by this hypothetical disease, one would need to wait for treatment. Furthermore, it was communicated to participants that on the request of the concerned health authority of their country, a committee of experts had proposed a number of alternative distributions of waiting times for people with this disease.

Our reason to make people who would become affected by the hypothetical disease to be those with some form of (permanent, temporary or self) employment is to emphasize that any deterioration in health due to the disease could have economic (i.e. income-related) consequences as well. The rationale behind targeting those between 30 and 50 years old is also to focus on the most economically productive age group to whom the opportunity cost of not being able to work (and possibly also childcare and/or informal care) while waiting for treatment would be considered significant. We asked Group two participants about a person of opposite gender to prevent them from assuming that the disease could affect them as well. Besides, we asked participants from this group to picture a close relative or friend so that they (presumably) made decisions as cautiously as they would have made them for themselves. Therefore, following the framework of perspectives when eliciting preferences in health proposed by [20], the scenario presented to Group one participants aligned with the socially inclusive personal perspective to social decision making in healthcare, as it concerns both the respondent and others, whereas the scenario presented to Group two participants aligned with the social perspective, as it concerns people other than the respondent.

### Choice experiment design

We used two types of designs: “Design A”, where participants in Group one (Group two) were not informed and, hence, did not know their (their close relative’s or friend’s) position in/the distribution of waiting times if they (their close relative or friend) become affected by the disease, and “Design B” where participants received this information. The first design mimics a design in which participants make choices behind a “veil of ignorance”: participants do not know their (close relative’s or friend’s) position in the distribution of waiting times albeit they have information that they (their close relative or friend) are part of the group that may become affected.

The choice tasks in both designs were composed of two alternatives, each representing a distribution of treatment waiting times over patients that would become affected by the hypothetical disease. In each alternative, the waiting times range from 3 to 15 weeks, i.e. an affected person would not be able to receive treatment before 3 weeks and would not wait for treatment for more than 15 weeks after being diagnosed with the disease. More specifically, the waiting times in each alternative (of each choice task) were 3, 6, 9, 12 and 15 weeks. However, the distribution of waiting times across patients differs between alternatives in a choice task, i.e. the share of patients who would receive treatment after a specific waiting time differed between alternatives in a choice task.

First, we generated an efficient design consisting of 15 choice tasks using the experimental design software Ngene [23]. The design generates different combinations of waiting times in weeks, e.g. (3, 6), (9, 12), (15, 6), etc. In each combination, the first (second) entry denotes the waiting time in the first (second) alternative. Each choice task presented to the participants, however, shows all waiting times (i.e. 3, 6, 9, 12 and 15 weeks) in each alternative, in combination with the respective shares of patients to whom a specific waiting time applies. Waiting time combinations generated by the design, which have sufficient variation across choice tasks, were subsequently used to systematically vary the distribution of waiting times across alternatives in a choice task. More specifically, we assigned distribution weights (that represent the share of patients) to the different waiting times in each alternative in such a way that one of the two alternatives appears to indicate a preference aligned to a positional concern. That is, the positional alternative shows a longer waiting time (than the waiting time indicated in the non-positional alternative) in absolute terms, but a shorter waiting time relative to a large proportion of other affected people (see Fig. 2).

Therefore, we created variations across choice tasks in three dimensions, namely, levels of waiting times (which enable identification of the SI component), relative waiting times (which enable identification of the PC component) as well as distributions of waiting times (which enable identification of the DC component). To ensure that our design enables (efficient) identification of model parameters from actual choice data, we test the final design with simulated choices by artificial decision makers. The results from this simulation exercise are presented in Appendix A1. It can be seen from Figs. 4 and 5 of Appendix A1 that our design enables true values of parameters to be recovered with high precision even with a small number of choices, supporting the efficiency of the design.^3^

**Fig. 1:**
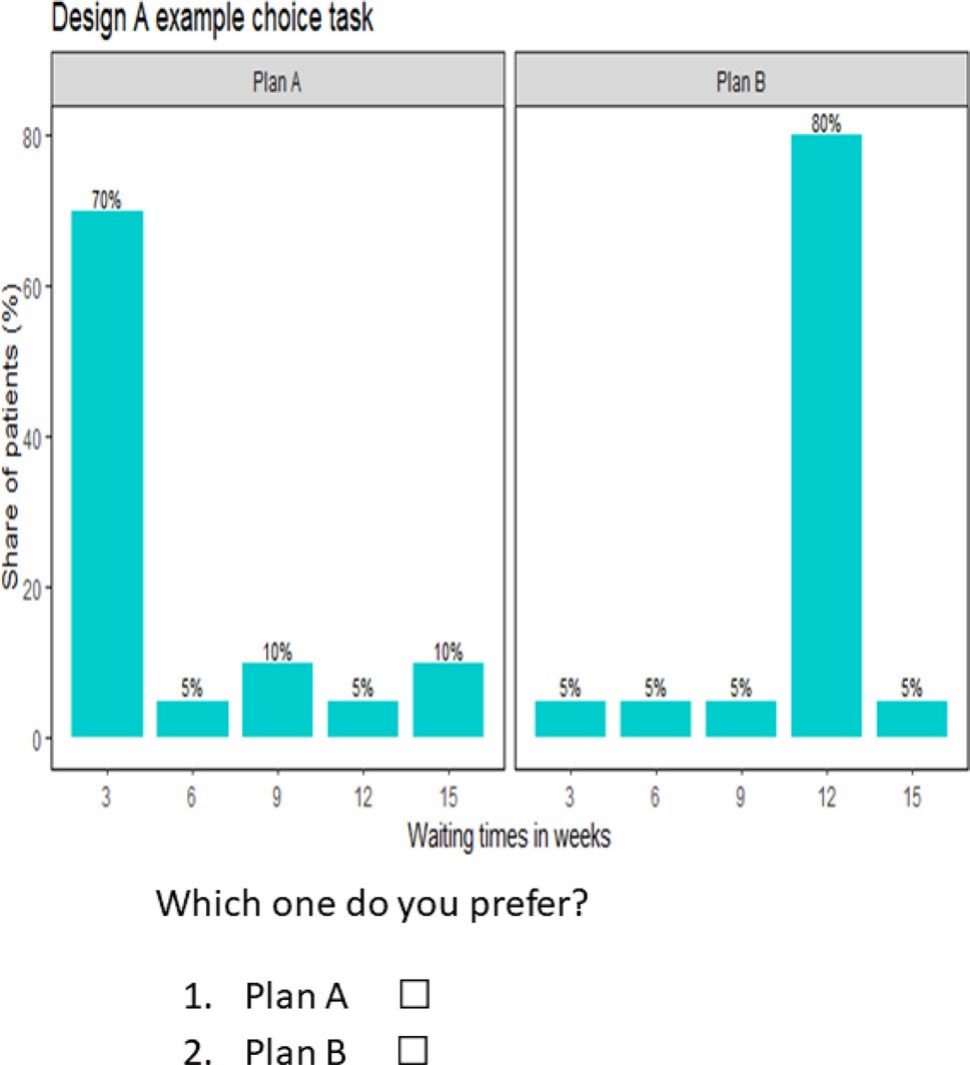
Example choice task for Design A

**Fig. 2:**
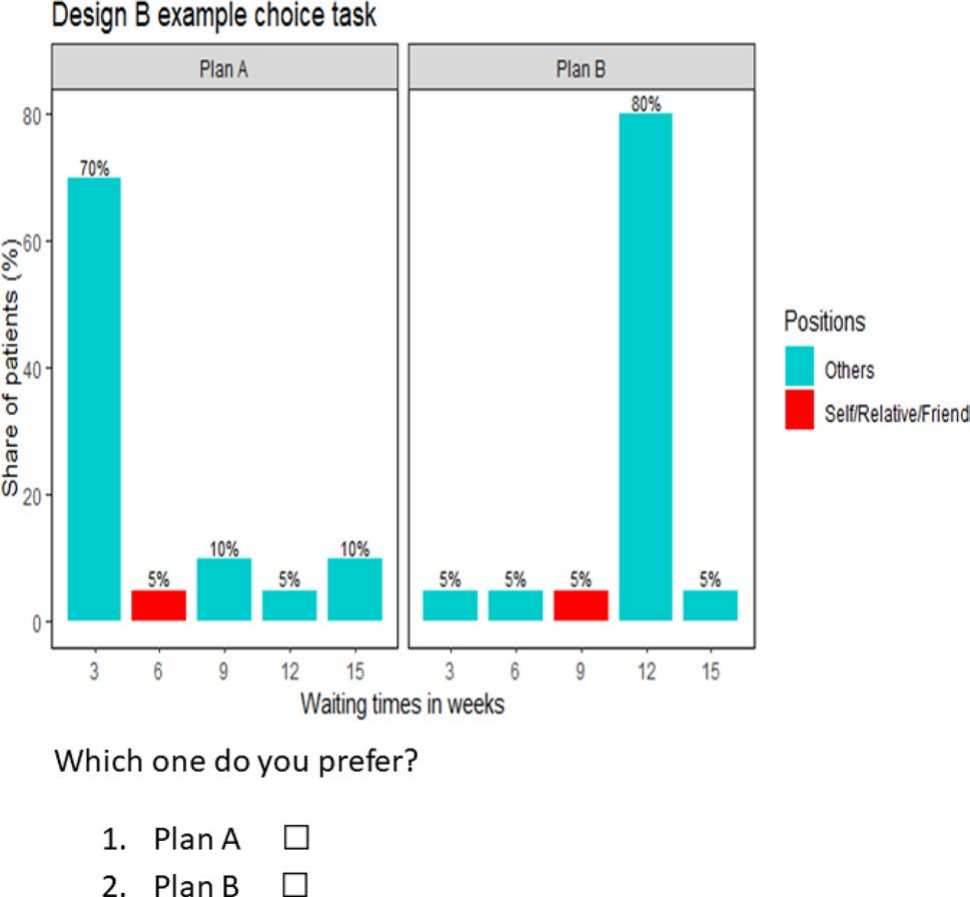
Example choice task for Design B

We note that the only difference between Design A and Design B is that in Design A participants are not informed about their (their close relative’s or friend’s) position in the distribution of waiting times (see example, choice tasks in Fig. 1), while this is indicated for participants facing Design B with a “red” colour (see Fig. 2). For this reason, only Design B can be used to identify the relative importance of the three motivations. A complete list of Design B choice tasks for Group one is available in Appendix A2.

Figures 1 and 2, respectively, show the distributions of waiting times in Design A and Design B. In both designs, the distributions vary between alternatives, i.e. Plan A and Plan B. While participants do not know about their (close relative’s or friend’s) position in the distribution of waiting times in Fig. 1 (Design A), this information is indicated by the red colour for participants in Fig. 2 (Design B).

In Table 1, we summarize the Design–Group combinations for the choice experiment.

**Table 1:** Description of 2 × 2 Design–group combinations

Design	Group	
	Group one (socially inclusive personal perspective)	Group two (social perspective)
Design A (veil of ignorance)	Participants make choices for self with no information about their position in the distribution of waiting times	Participants make choices for a close relative or friend with no information about the close relative’s or friend’s position in the distribution of waiting times
Design B (fully informed)	Participants make choices for self with information about their position in the distribution of waiting times	Participants make choices for a close relative or friend with information about the close relative’s or friend’s position in the distribution of waiting times

### Data

The survey questionnaire contains three sections. In the first section, participants (in both Group one and Group two) were asked about their basic socio-demographic information. Based on this information, participants were screened for eligibility to participate. In the second part of the survey, eligible participants were presented with the EuroQol EQ-5D-5L instrument. Participants from Group one were asked to rate their own current health state by means of this instrument, while participants from Group two were asked to rate their close relative’s or friend’s current health state using an adapted version of the instrument reflecting the proxy rating in the domain level descriptions. The final section of the survey contains the above choice experiment scenario in which participants completed two different series of choice tasks corresponding to Designs A and B, and debriefing questions about their choices.

Participants in both groups completed a series of 5 choice tasks based on Design A, followed by a series of 15 choice tasks based on Design B, always in that order as Design B provided additional information. Our reason to restrict the number of choice tasks in Design A to 5 is to reduce fatigue from answering the 15 choice questions in Design B, where we anticipate participants may require more deliberation. The choice tasks within each design were presented in random order to minimize the possibility of participants cross-checking choice tasks from Design A and Design B.

Participants were recruited from the US and the UK panels of the online survey company Dynata, and the survey was administered online in October 2021.^4^ The total number of Group one participants was 1552, of whom 769 (49.5%) were from the US and 783 (50.5%) were from the UK. Group two participants were 1540 in total, 773 (50.2%) from the US and 767 (49.8%) from the UK. For a summary of the sample characteristics, see Appendix A3.

## Modelling approach

Suppose ${U}_{\mathrm{nit}}$ denotes the utility decision maker $n$ derives from alternative $i$ with $K$ characteristics in choice situation $t$. Following the random utility maximization framework [24], the decision maker is assumed to choose the alternative that overall performs best or maximizes utility, which is composed of an observable, deterministic component and a random unobservable component. Using a linear-in-parameters additive utility specification for the observed component, ${U}_{\mathrm{nit}}$ can be expressed as

1 $${U}_{\mathrm{nit}}={\sum }_{k}^{K}{\beta }_{k}{x}_{\mathrm{nikt}}+{\varepsilon }_{\mathrm{nit}},$$

where ${x}_{\mathrm{nikt}}$ represents the level of alternative $i$’s characteristic $k$ in choice task $t$, ${\beta }_{k}$ is the weight corresponding to $k$ which is to be estimated, and ${\varepsilon }_{\mathrm{nit}}$ is a random error term with an extreme value Type I distribution.

Given this framework, we start with explaining the analysis approach we use to understand the extent to which disclosing information about one’s position in a distribution (i.e. in our case, waiting time) influences choices. When a decision maker’s (close relative’s or friend’s) position is disclosed, the observed component of utility for an alternative is a function of the waiting times and the shape of its distribution (that can be summarized by a distribution metric). Therefore, we augment the utility specification in Eq. (1) with a distributional component, which is the second term on the right-hand side of Eq. (2). Note that the number of characteristics $K$ in our application is one (i.e. waiting time), and hence we do not need the summation operator here. Then,

2 $${U}_{\mathrm{nit}}={\beta }^{SI}{x}_{\mathrm{nit}}+{\beta }^{DC}{D}_{it}+{\varepsilon }_{\mathrm{nit}},$$

where ${x}_{\mathrm{nit}}$, with a corresponding parameter ${\beta }^{\mathrm{SI}}$, is the decision maker’s (close relative’s or friend’s) waiting time. In Eq. (2), ${\beta }^{\mathrm{DC}}$ is a parameter corresponding to ${D}_{it}$, which is the value of any considered distribution metric in alternative $i$ of choice task $t$. As indicated in the introduction, we considered several metrics to represent DC and explain the decision makers’ choice between alternative waiting time distributions. The metrics we considered include the expected value, minimax, Shannon's entropy and the generalized entropy (GE) inequality index. We define the expected waiting time values as the weighted average of waiting times for an alternative and minimax waiting time values as the proportion of patients with the longest possible waiting time (i.e. 15 weeks) in an alternative. The latter captures distributional preferences focusing on the most disadvantaged ones; in our case, on the share of patients with the longest waiting time. Shannon’s entropy gives emphasis just to equality, while the GE inequality index is sensitive to longer waiting times when its sensitivity parameter α is high (e.g. α = 10) and lower waiting times when α is low (e.g. α = 0). The formulas used to compute the metrics are provided in Appendix A4.

We dummy coded alternatives based on the value of each distribution metric we consider to capture DC. Specifically, depending on the considered metric, DC takes a value of 1 (i.e. ${D}_{it}=1$) if an alternative has the lowest expected value, lowest minimax value, lowest Shannon's entropy value or smallest GE inequality index in a choice task, and a value equal to 0 (i.e. ${D}_{it}=0$) in all other cases. Similarly, we replaced ${x}_{\mathrm{nit}}$ in Eq. (2) by an indicator which takes a value of $1$ if $i$ represents the alternative with the lower waiting time in choice task $t$ and a value of $0$ otherwise. We find that dummy coding of alternatives instead of using actual levels of waiting times and distributional metrics not only makes comparing the parameters associated with the different motivations more straightforward, but also improves stability in estimation of model parameters. In addition, compared to models estimated based on actual levels, dummy coding values produce better model fit and qualitatively similar results.

Alternatively, when a decision maker’s (close relative’s or friend’s) position is not disclosed (i.e. under a “veil of ignorance”), the choice between alternative plans depends only on the shape of the distribution of waiting times. In this case, only ${\upbeta }^{\mathrm{DC}}$ can be estimated. Therefore, the effect of disclosing the decision maker’s (close relative’s or friend’s) position on choices can be examined by comparing between ${\upbeta }^{\mathrm{DC}}$ estimated on observations with (i.e. Design B) and without (i.e. Design A) this information.

Subsequently, the utility function for the model that accommodates PC and DC simultaneously with SI is specified as

3 $${U}_{\mathrm{nit}}={\beta }^{\mathrm{SI}}{S}_{\mathrm{nit}}+{\beta }^{\mathrm{PC}}{P}_{\mathrm{nit}}+{\beta }^{\mathrm{DC}}{D}_{it}+{\varepsilon }_{\mathrm{nit}},$$

where ${\mathrm{S}}_{\mathrm{nit}}$, ${P}_{\mathrm{nit}}$ and ${D}_{it}$, with corresponding parameters ${\upbeta }^{\mathrm{SI}}$, ${\beta }^{\mathrm{PC}}$ and ${\upbeta }^{\mathrm{DC}}$, respectively, are indicators for SI (i.e. whether or not $i$ is the alternative with the lower waiting time in $t$), PC (i.e. whether or not $i$ represents the positional alternative in $t$) and DC (i.e. whether or not $i$ is the alternative with the a lower inequality, lower expected waiting time or lower minimax value in $t$) in choice task $t$.

**Fig. 3:**
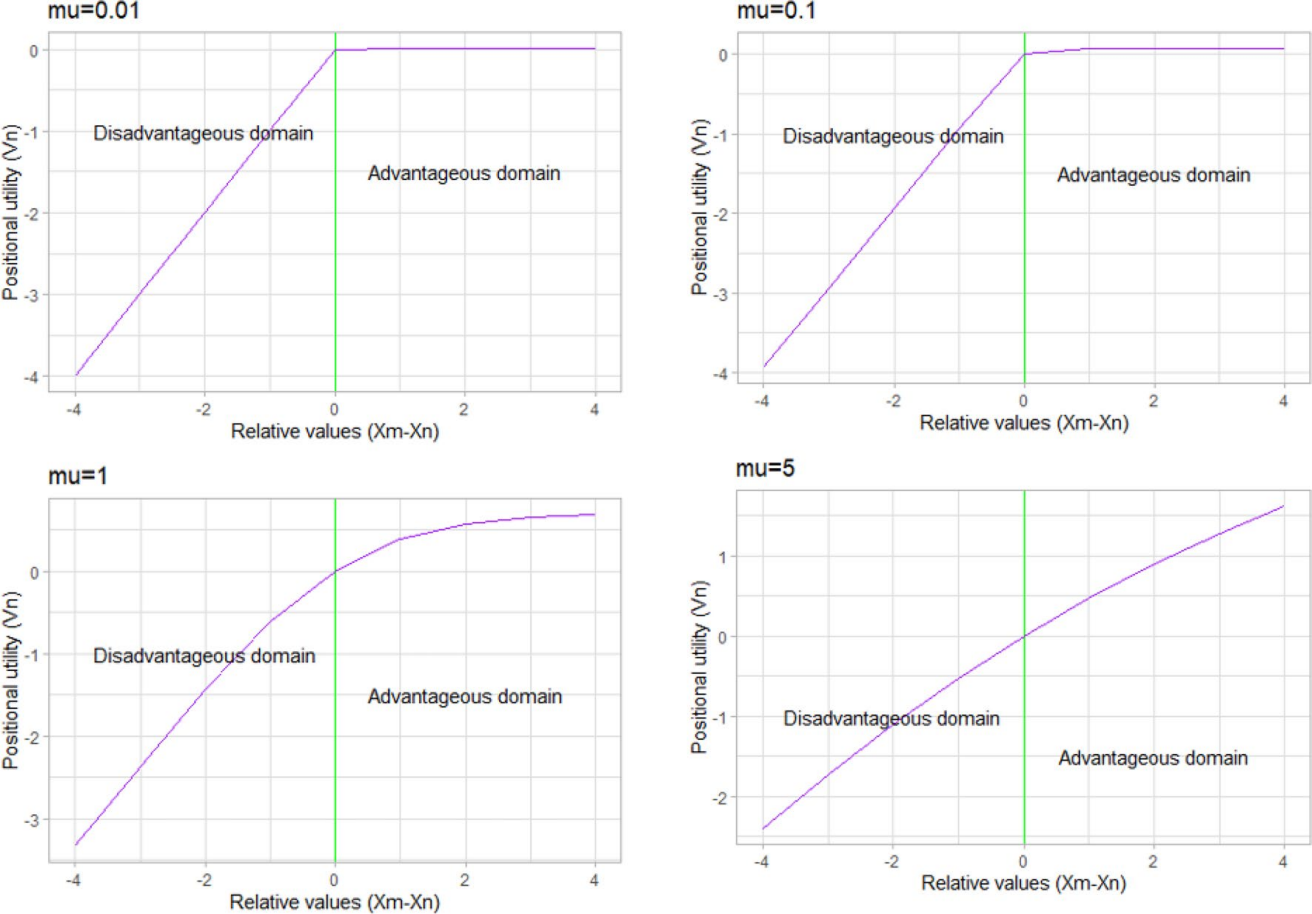
Positional utility of individual $n$ (i.e. Relative to individual m) for $\beta =-1$ and different values of $\mu$

**Fig. 4:**
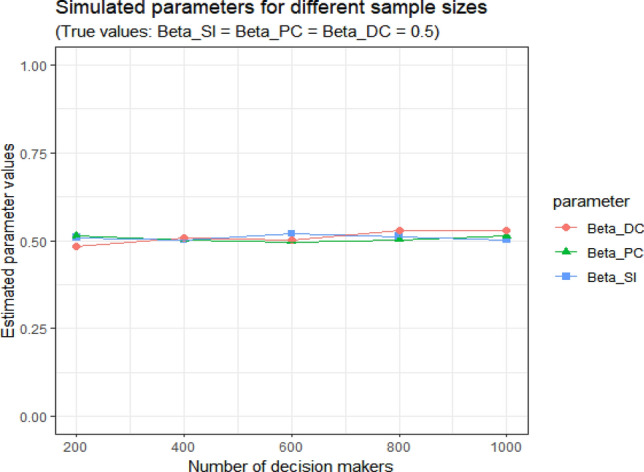
Parameter estimates from different number of simulated choices

**Fig. 5:**
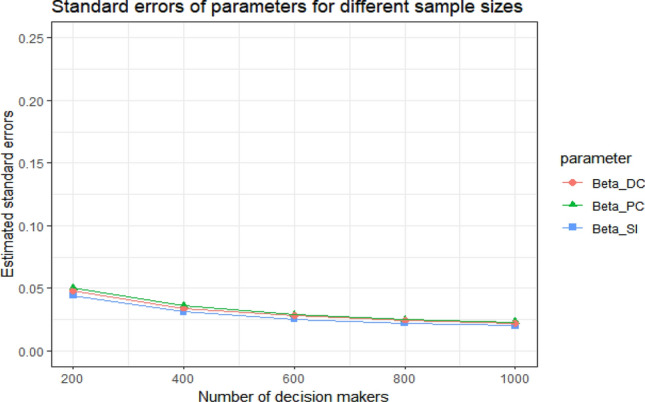
Standard errors of parameter estimated for different number of simulated choices

Finally, to test for potential asymmetry between an advantageous inequality and a disadvantageous inequality in waiting time of equal magnitude, we adapt a variant of the random regret minimization (RRM) model, $\mu RRM$ model, explained in van Cranenburgh et al. [25]. More specifically, we use the following specification of a relative utility function:

4 $${U}_{\mathrm{nit}}=-{\sum }_{m\ne n}\mu \left(\mathit{ln}\left(1+\mathrm{exp}\left(\frac{\beta }{\mu }{\omega }_{\mathrm{mit}}\left({x}_{\mathrm{mit}}-{x}_{\mathrm{nit}}\right)\right)\right)-ln(2)\right)+{\varepsilon }_{\mathrm{nit}},$$

where ${x}_{\mathrm{nit}}$ (${x}_{\mathrm{mit}}$) is individual $n$’s ($m$’s) waiting time in alternative $i$ of choice task $t$, ${\omega }_{\mathrm{mit}}$ represents the proportion of patients with waiting time ${x}_{\mathrm{mit}}$ and $\beta$ denotes the marginal utility of relative waiting time. The parameter $\mu$ informs about the degree of asymmetry between advantageous and disadvantageous inequalities of equal magnitude. For a given value of $\beta$ ($\beta =-1$), Fig. 3 depicts what the deterministic component in Eq. (4) looks like for different values of $\mu$ over a range of relative values (i.e. ${x}_{m}-{x}_{n}$). It is evident from Fig. 3 that as the value of $\mu$ increases, the deterministic utility from an advantageous inequality (to the right of the vertical green line) and disutility from a disadvantageous inequality of equal magnitude (to the left of the vertical green line) becomes more and more symmetric with respect to 0 (where the vertical green line intersects the utility curve). Colloquially put, as $\mu$ becomes smaller, the disutility that a decision maker (patient) would derive from the notion that others have shorter waiting times is greater than the utility she/he would derive from the notion that others have longer waiting times.

Assuming i.i.d. extreme value Type I error terms, we calculate the probability that $n$ chooses alternative $i$ in choice occasion $t$ for each model using the logit formula as follows:

5 $$\mathit{Pr}\left({i}_{\mathrm{nt}}|{x}_{\mathrm{nit}}\right)=\frac{\mathrm{exp}\left({V}_{\mathrm{nit}}\right)}{\sum_{j=1}^{J}\mathrm{exp}\left({V}_{\mathrm{njt}}\right)},$$

where ${V}_{\mathrm{nit}}$ denotes the deterministic component of any of the utility functions.

The probability of a sequence $T$ choices by $n$, ${y}_{n}={i}_{n1}, {i}_{n2}, \dots \dots {i}_{nT}$, is then computed as

6 $$Pr\left( {y_{n} |x_{{{\text{int}}}} } \right) = \prod\limits_{{{t = 1}}}^{T} {} Pr\left( {i_{{{\text{nt}}}} |x_{{{\text{nit}}}} } \right).$$

## Empirical results

In this section, we present the empirical results of the choice experiment explained in Sect. "Choice experiment design and data" (see Table 1 for the Design–Group combinations). Before an in-depth model-based analysis, we provide a descriptive assessment of the observed choices of respondents for both groups.

### Descriptive analysis of choices

As a first descriptive analysis, Table 2 summarizes the extent to which choices of respondents are in line with SI, PC and DC for the different sub-samples. More specifically, the table shows the average number of times (out of 15 choice tasks in Design B) respondents make their choice in line with each motivation for each sub-sample. We considered a choice of an alternative as consistent with SI if the chosen alternative represents a shorter waiting time compared to another alternative. Also, we define a choice for an alternative with a lower proportion of patients corresponding to waiting times shorter than the respondent’s (relative or friend) waiting time (again relative to the other alternative) is in line with PC. Similarly, we considered a choice for an alternative with lower expected waiting time, Shannon entropy, GE index and smaller proportion of patients corresponding to a waiting time of 15 weeks (relative to another alternative) as consistent with inequality aversion/fairness. To distinguish between the different sub-samples, we denote Group one respondents from the US (UK) by US-1 (UK-1) and Group two respondents from the US (UK) by US-2 (UK-2). We observe that for all the sub-samples, respondents’ choices are consistent with SI, PC and DC in more than half of the cases, suggesting the importance of all these three motivations for decision making. In terms of individual motivations, within countries, choices of respondents from the US are more consistent with SI than with the other motives (see higher mean scores in column “SI” for US-1 and US-2 in Table 2), while UK respondents’ choices are aligned more to DC (based on a minimax principle) than to the other motives.

**Table 2:** Average number of times respondents make choices consistent with a motivation

Sub-sample	Motivation for decision making							
	SI	PC	Metrics for distribution considerations (DC)					
			Expected value	Minimax	GE-$\alpha$ = 10	GE-$\alpha$ = 1	GE-$\alpha$ = 0	Entropy
US-1	9.1	8.0	8.2	8.8	8.2	8.3	8.2	8.3
UK-1	9.5	7.7	9.1	9.6	9.1	9.1	8.9	8.2
US-2	9.5	7.9	8.8	9.3	8.8	8.8	8.7	8.4
UK-2	9.6	7.7	9.3	9.8	9.3	9.3	9.1	8.2
*p* values of differences								
US-1 vs. UK-1	0.0001	0.0041	0.0000	0.0000	0.0000	0.0000	0.0000	0.3182
US-2 vs. UK-2	0.7437	0.0455	0.0017	0.0017	0.0017	0.0021	0.0131	0.0957
US-1 vs. US-2	0.0001	0.3539	0.0005	0.0003	0.0005	0.0003	0.0003	0.6796
UK-1 vs. UK-2	0.7370	0.9474	0.2703	0.2225	0.3956	0.1864	0.3956	0.7643

Note: Group one respondents from the US (UK) are denoted by US-1 (UK-1), while Group two respondents from the US (UK) are denoted by US-2 (UK-2). *p* values of differences show that the t test results on whether the average number of times (out of 15) respondents make choices consistent with a motivation significantly vary between different sub-samples. For example, “0.0001” corresponding to US-1 vs. UK-1 and SI, indicates t test p value from comparing the average number of times Group 1 respondents choose the alternative (plan) with the lower waiting time in the US with the UK

Comparing between respondents who chose for themselves (i.e. Group one) and those who choose for their close relative or friend (i.e. Group two), Table 2 shows that the latter group significantly more often chose alternatives representing SI and DC in the US (see p values for US-1 vs US-2 corresponding to columns “SI” and “DC”). This is not the case for UK respondents (see *p* values for UK-1 vs. UK-2).

When we look at differences between the two countries, we observe that UK-1 respondents made choices consistent with SI as well as DC significantly more often than US-1 respondents (see p-values for US-1 vs UK-1 and US-2 vs UK-2 corresponding to columns “SI” and “DC”). Also, we notice that UK-2 respondents chose in line with DC significantly more often than those from the US-2. Furthermore, respondents from the US chose the alternative representing a positional concern significantly more often than respondents from the UK (see p values for US-1 vs UK-1 and US-2 vs UK-2 corresponding to column “PC”). Among the distributional metrics we considered, using the minimax principle generates the highest average number of choices consistent with DC. However, this observation does not take into account the potential influence of information about the decision maker’s (close relative’s or friend’s) waiting times on distributional preferences, which we examine in the next section.

### Discrete choice model estimation results

This section presents the results of the experiment based on estimation of a series of binary choice models. We first elaborate on the suitability of the different metrics considered to represent DC. Next, we provide results in which we compare Design A with Design B, that is, observed choices made without information about the decision maker’s (close relative’s or friend’s) position in the waiting time distribution (i.e. choices from behind a “veil of ignorance”) and choices made with this information. This will be followed by a presentation of our results regarding decision makers’ evaluation of advantageous and disadvantageous inequalities of equal magnitude. Finally, we offer a series of binary logit model results where we compare the decision weight for each motivation between Group one and Group two respondents as well as between respondents from the US and the UK.

#### Selection of a distribution metric

Instead of a priori selecting and using a single distributional metric, the approach we followed is to test a number of different metrics and select the one that is most suitable to the data we have at hand based on model fit criteria. Accordingly, we estimated Eq. (2) where we use each of the aforementioned metrics for the distributional component on observations based on Design B, for each sub-sample (i.e. US-1, US-2, UK-1 and UK-2). Comparing the results based on final log-likelihood value, we find that expected value of waiting time is the most suitable to summarize DC and explain our data. The result is robust to estimation sample (see Table 3).

**Table 3:** A comparison of different distributional metrics to summarize DC

Distribution metric	Parameter	US-1	UK-1	US-2	UK-2
		Est. (*t* ratio)	Est. (*t* ratio)	Est. (*t* ratio)	Est. (*t* ratio)
Expected value	${\beta }^{\mathrm{DC}}$	0.162 (8.5)	0.406 (21.0)	0.309 (16.0)	0.460 (20.5)
	${\beta }^{\mathrm{SI}}$	0.289 (14.6)	0.407 (20.4)	0.414 (20.7)	0.417 (20.5)
	Log-likelihood	− 7831.6	− 7647.4	− 7641.9	− 7416.4
Minimax	${\beta }^{\mathrm{DC}}$	0.164 (8.3)	0.406 (20.1)	0.311 (15.4)	0.466 (22.8)
	${\beta }^{\mathrm{SI}}$	0.276 (13.8)	0.376 (18.7)	0.391 (19.4)	0.380 (18.6)
	Log-likelihood	− 7833.7	− 7711.2	− 7651.9	− 7433.7
GE-$\alpha$ = 10	${\beta }^{\mathrm{DC}}$	0.162 (8.5)	0.406 (21.0)	0.309 (16.0)	0.460 (20.5)
	${\beta }^{\mathrm{SI}}$	0.289 (14.6)	0.407 (20.4)	0.414 (20.7)	0.417 (20.5)
	Log-likelihood	− 7831.6	− 7647.4	− 7641.9	− 7416.4
GE-$\alpha$ = 1	${\beta }^{\mathrm{DC}}$	0.145 (7.5)	0.351 (17.9)	0.271 (13.8)	0.410 (20.6)
	${\beta }^{\mathrm{SI}}$	0.269 (13.3)	0.359 (17.6)	0.378 (18.5)	0.358 (17.4)
	Log-likelihood	− 7840.1	− 7711.2	− 7676.4	− 7483.8
GE-$\alpha$ = 0	${\beta }^{\mathrm{DC}}$	0.084 (4.1)	0.257 (12.5)	0.184 (8.9)	0.291 (14.0)
	${\beta }^{\mathrm{SI}}$	0.273 (12.8)	0.343 (16.1)	0.373 (17.4)	0.342 (15.9)
	Log-likelihood	− 7859.5	− 7793.9	− 7732.2	− 7599.4
Entropy	${\beta }^{\mathrm{DC}}$	− 0.104 (-5.1)	− 0.169 (− 8.2)	− 0.110 (− 0.1)	− 0.183 (− 8.7)
	${\beta }^{\mathrm{SI}}$	0.325 (16.4)	0.475 (23.8)	0.466 (23.3)	0.490 (24.2)
	Log-likelihood	− 7855.0	− 7838.5	− 7757.1	− 7658.8

Note: Group one respondents from the US (UK) are denoted by US-1 (UK-1), while Group two respondents from the US (UK) are denoted by US-2 (UK-2). The parameters ${\beta }^{\mathrm{SI}}$ and ${\beta }^{\mathrm{DC}}$, respectively, indicate the weight for SI and DC. Data used to estimate ${\beta }^{\mathrm{SI}}$ in a sub-sample do not vary across the distribution metrics, but ${\beta }^{\mathrm{DC}}$ is estimated with data generated using a corresponding distribution metric indicated in the first column

#### Distributional preferences from behind the veil of ignorance versus with full information about one’s own position

Comparing the design where respondents do not know information about their (close relative’s or friend’s) position in the distribution of waiting times (i.e. Design A) with the design where this information is clearly indicated (i.e. Design B) enables us to examine whether and how (i.e. positively or negatively) disclosing this information influences distributional preferences. To this end, we estimate Eq. (2) on observations obtained using Design A and on observations obtained using Design B separately for each sub-sample (i.e. US-1, US-2, UK-1 and UK-2). Since specific waiting times are not indicated, we estimate Eq. (2) without the ${\beta }^{\mathrm{SI}}$ parameter for Design A. Therefore, in each sub-sample, we compare the parameters corresponding to the weights for DC (i.e. ${\beta }^{\mathrm{DC}}$) between the two designs. For estimation, alternatives in each choice task are dummy coded as either or not representing a distributional concern in a choice task (i.e. $\mathrm{DC}=1$ otherwise $\mathrm{DC}=0$).

The DC metric used for the results given in Table 4 is the expected value, which, as shown in the preceding sub-section, is found to provide the best explanation to our data. Our results reported in Table 4 show that decision makers informed about their (close relative’s or friend’s) position in the distribution of waiting times (i.e. Design B) attach significantly smaller weight to DC as compared to respondents who do not have this information (i.e. Design A): for all the sub-samples, ${\beta }^{\mathrm{DC}}$ corresponding to Design B is significantly smaller than ${\beta }^{\mathrm{DC}}$ corresponding to Design A. In other words, presenting participants with information about their own position in the distribution of waiting times diminishes their distributional preferences, but does not eliminate them.

**Table 4:** Estimation results for distributional preferences

Sub-sample	Design	Parameters		Model log-likelihood	Observations
		${\beta }^{\mathrm{SI}}$ Est. (*t-ratio*)	${\beta }^{\mathrm{DC}}$ Est. (*t-ratio*)		
US-1	Design A		0.397 (12.1)	− 2590.9	3845
	Design B	0.162 (8.5)	0.289 (14.6)	− 7831.6	11,535
UK-1	Design A		0.759 (22.1)	− 2450.6	3915
	Design B	0.406 (21.0)	0.407 (20.4)	− 7647.4	11,745
US-2	Design A		0.578 (17.2)	− 2524.1	3865
	Design B	0.309 (16.0)	0.414 (20.7)	− 7641.9	11,595
UK-2	Design A		0.779 (22.4)	− 2387.8	3835
	Design B	0.460 (20.5	0.417 (20.5)	− 7416.4	11,505

Note: Group one respondents from the US (UK) are denoted by US-1 (UK-1), while Group two respondents from the US (UK) are denoted by US-2 (UK-2). The parameters ${\beta }^{\mathrm{SI}}$ and ${\beta }^{\mathrm{DC}}$, respectively, indicate the weight for SI and DC. ${\beta }^{\mathrm{SI}}$ cannot be estimated for Design A, since the respondent’s (relative’s or friend’s) waiting times were not indicated in this design

#### Advantageous and disadvantageous inequality

Under this sub-section, we present our analysis of potential asymmetry between advantageous and disadvantageous inequalities of equal magnitude, which is useful to understand the implications of a redistribution of waiting times on overall well-being. For this purpose, we estimate Eq. (4) on data obtained using Design B. As we graphically illustrate in Fig. 3, the degree of asymmetry between advantageous and disadvantageous inequality is closely tied with the value of $\mu$. In our analysis, we consider different values for $\mu$ ranging from 0.01 to 5 and compare the estimation results based on model fit.

The estimation results we obtained for all the sub-samples indicate that model fit improves when we take a larger value for $\mu$. In particular, improvements in final log-likelihood value become more and more insignificant as $\mu$ gets larger, and the gain is negligible for values closer to and above a value of 5. This result suggests that there is limited to no asymmetry in respondents’ evaluations of advantageous and disadvantageous inequality. For reasons of space, we provide the estimation results in Appendix A5.

#### Relative importance of self-interest, positional concerns and distributional considerations

In this section, we examine whether and to what extent each individual motivation explains choices. To this end, we use Design B and estimated Eq. (3) where we included only the indicator for a considered motivation as an explanatory variable. We then proceed with exploring how different combinations of motivations for decision making explain respondents’ observed choices. Finally, we present the results where we included all the three motivations simultaneously. Table 5 summarizes the results for all these estimations.

**Table 5:** Relative importance of self-interest (SI), positional concerns (PC) and distributional considerations (DC) in decision making using Design B

Group	Parameters and final log-likelihood	Motivations for decision making											
		SI		PC		SI + PC		SI + DC (expected)		PC + DC (expected)		SI + PC + DC (expected value)	
		US	UK	US	UK	US	UK	US	UK	US	UK	US	UK
		Estimate (*t* ratio)	Estimate (*t* ratio)	Estimate (*t* ratio)	Estimate (*t* ratio)	Estimate (*t* ratio)	Estimate (*t* ratio)	Estimate (*t* ratio)	Estimate (*t* ratio)	Estimate (*t* ratio)	Estimate (*t* ratio)	Estimate (*t* ratio)	Estimate (*t* ratio)
Group one	${\beta }^{SI}$	0.310 (15.9)	0.448 (22.9)			0.301 (14.1)	0.525 (23.9)	0.289 (14.6)	0.407 (20.4)			0.194 (8.3)	0.335 (14.1)
	${\beta }^{PC}$			0.140 (7.5)	0.043 (2.3)	0.021 (1.0)	− 0.172 (− 8.1)			0.307 (14.0)	0.350 (15.4)	0.193 (7.4)	0.148 (5.4)
	${\beta }^{DC}$							0.162 (8.5)	0.406 (21.0)	0.346 (15.8)	0.620 (27.2)	0.267 (11.2)	0.487 (19.8)
	Final log-likelihood	− 7867.9	− 7872.1	− 7967.2	− 8138.3	− 7867.4	− 7838.5	− 7831.6	− 7647.4	− 7838.3	− 7733.7	− 7803.8	− 7632.4
	Observations	11,535	11,745	11,535	11,745	11,535	11,745	11,535	11,745	11,535	11,745	11,535	11,745
Group two	${\beta }^{SI}$	0.449 (22.8)	0.461 (23.2)			0.487 (22.3)	0.542 (24.3)	0.414 (20.7)	0.417 (20.5)			0.326 (13.8)	0.323 (13.4)
	${\beta }^{PC}$			0.109 (5.8)	0.041 (2.2)	− 0.087 (− 4.1)	− 0.181 (− 8.5)			0.376 (16.6)	0.389 (16.5)	0.181 (6.7)	0.194 (7.0)
	${\beta }^{DC}$							0.309 (16.3)	0.460 (23.5)	0.539 (23.8)	0.696 (29.6)	0.408 (16.6)	0.568 (22.4)
	Final log- likelihood	− 7771.4	− 7697.3	− 8020.0	− 7972.3	− 7762.9	− 7660.9	− 7641.9	− 7416.4	− 7715.8	− 7483.8	− 7619.3	− 7392.0
	Observations	11,595	11,505	11,595	11,505	11,595	11,505	11,595	11,505	11,595	11,505	11,595	11,505

First, we observe that both SI and PC, independently, are significant in explaining choices of both groups of respondents from both the US and the UK. The explanatory power of SI is stronger than PC (see columns “SI” and “PC”). Second, when we combine PC with only SI (see column SI + PC), the weight for SI becomes larger, while the coefficient associated with PC turns negative for both groups from the UK and for the second group from the US. A potential explanation for this is that SI (which usually overlaps with DC) also captures part of the variation explained by DC to which respondents attach the greatest weight. Third, the results where we combine each of these two motivations with DC (based on expected value) also indicate that the parameters associated with each of the motivations are significant in all sub-samples (see columns “SI + DC” and “PC + DC”). Judging by model fit (i.e. final log-likelihood value), the model that combines SI with DC explains the data from each sub-sample significantly better than models combining PC with DC and PC with SI. This result suggests that, while it is a significant predictor of individual choice behaviour, particularly in our context, PC is not as strong as SI and DC, and leaving DC out of the equation could be misleading. The result here is consistent with the descriptive analysis presented in Sect. "Descriptive analysis of choices", where we see a relatively lower average number of choices consistent with PC.

Finally, when including the three motivations simultaneously (see column “SI + PC + DC”), we find that all the three motivations are significant in explaining respondents’ choices irrespective of Group or country, that is, no matter who these choices affect (i.e. the decision makers or their close relative or friend) and regardless of where the decision makers live (i.e. the US or the UK). Participants in general attach the largest weight to how waiting times are distributed across potential patients (i.e. DC) followed by their own (or their close relative’s or friend’s) waiting time (i.e. SI). Beyond behavioural plausibility, our result shows that following this holistic approach offers a statistically superior model that is robust to empirical choice data collected under different perspectives (i.e. socially inclusive personal and social) and from two different countries (the results of which we explain in the subsequent sections). It is important to note that the comparisons we present here are based on models that do not attempt to disentangle potential differences in scale of utility (or variance of unobserved factors) versus differences in preference weights associated with each considered motivation. It is easily seen why, for the purpose of this study, such a de-composition of a total effect (difference) into its subcomponents (preference and scale differences) would be irrelevant: our analyses are aimed at identifying whether a particular motivation is more, or less, important in one experimental treatment compared to another one, or in one sample compared to another one. With ‘the importance of a motivation’ we refer to the extent to which it influences or determines choice behaviour. If we test whether, say, motivation X is a stronger determinant of choice behaviour in treatment (or sample) A than in treatment (or sample) B, we are not so much interested in testing to what extent any difference in importance is driven by a difference in the motivation’s importance relative to other observed motivations (also known as a preference difference), and to what extent it is driven by a difference in the motivation’s importance relative to other, unobserved factors (so known as a scale difference). Rather, we aim to empirically investigate whether in sum, the motivation plays a greater role in determining choice behaviour in sample or treatment A compared to B. However, for completeness, we have estimated models that control for potential scale differences by means of the Swait and Louviere [26] test. The estimation results for these models are available in Appendix A6 and show that the conclusions that can be drawn from them are qualitatively similar to the ones presented below.

#### Motivations for decision making: self vs. others

Understanding the relative importance of different motivations in health-related decisions that directly affect decision makers themselves (Group one) as compared to those that affect significant others (Group two) is useful to frame health interventions, when thinking of privately and publicly funded healthcare systems.

To start with similarities, the result given in Table 5 (under column “SI + PC + DC”) shows ranking by importance of motivations is identical between the two groups, for both countries. Nevertheless, we find that models estimated on observations from Group two are statistically superior to models estimated on corresponding Group one observations, and that Group two respondents attach significantly larger weight to DC. This is particularly true for respondents from the US where we see that ${\beta }^{\mathrm{DC}}$ as well as ${\beta }^{\mathrm{SI}}$ are significantly larger in Group two than in Group one. The result supports the idea that altruistic framing of decisions improves adoption of (health-related) behaviours that benefit others. We elaborate on this finding in light of literature on decision making for others in Sect. "Conclusions and discussion".

#### Comparison of motivations for decision making: US vs. UK

An additional important aspect of our study is that we conduct the empirical investigation on the relative importance of SI, PC and DC in decision making in two countries with very different healthcare systems. We use exactly the same choice experiment design for the US and UK samples. This enables us to associate any distinction between corresponding results not only to differences in healthcare systems but also to related cultural and political discrepancies. The results based on which we compare between the two countries are again given in Table 5 (under column “SI + PC + DC”).

Starting with Group one (i.e. participants making choices for self), Table 5 shows that respondents from the UK attach significantly larger weight to SI and DC than those from the US, which is consistent with the descriptive results. Although respondents from the US display relatively stronger PC than those from the UK, the difference is not statistically significant. Turning to Group two (i.e. participants making choices for a close relative or friend), we find that respondents from the UK place significantly larger weight on DC than their US counterparts. However, there is no significant difference between the two countries in terms of the weights associated with SI and PC. Importantly, as discussed in the previous section, the ranking of the three motivations by magnitude of respective weights remains the same for both countries: DC followed by SI and PC is the most important motivation in explaining respondents’ choices in both the US and the UK.

## Conclusions and discussion

This study presented empirical (experimental) evidence on the relative importance of self-interest, positional concerns and distributional considerations for decision making about access to healthcare services. Our study captures these three motivations together, which was enabled by a sophisticated, tailored-made experimental design. Our data comes from two countries (the UK and the US) with different healthcare systems and we frame participants in socially inclusive personal and social perspectives while varying their information level. In the experiment, participants were asked to make choices between alternative distributions of treatment waiting times for themselves or for a close relative or friend of opposite gender. Under both perspectives, participants were asked to indicate their preferred alternative when their position in the distribution of waiting times is clearly indicated and when this information is not provided, which mimics a choice behind a “veil of ignorance”. This design enabled us to efficiently identify the relative weights placed on each motivation (self-interest, positional concerns, and distributional considerations) for decision making. These features of the experiment distinguish our study from previous research efforts aimed at identifying the relative importance of various motivations for individual decision making, e.g. [17, 18].

We found that self-interest, positional concerns and distributional considerations jointly significantly explain choice behaviour related to the specific empirical context of treatment waiting time allocation. Our results show that distributional considerations, followed by self-interest and positional concerns, is the most important motivation, and this holds irrespective of the choice perspective (i.e. socially inclusive personal and social) and the country where decision makers live (i.e. the US or the UK). While our approach remains useful to decipher the relative importance of the three motivations for individual decision making, it behoves us to acknowledge that the exact order of importance of the motivations could be due to our particular design or empirical context. For this reason, a suggestion for future research would be to validate our results in other healthcare contexts. It is also important to emphasize that our experiment is based on a hypothetical (disease) scenario, and hence interpretation of the results should take this into account.

Our results are partly novel and partly confirm findings from previous research. For instance, we found that participants place a larger weight on the distribution of waiting times when they lack information about their (close relative’s or friend’s) position in the distribution of waiting times (i.e. when choosing behind a “veil of ignorance”) than when they have access to such information. This result is in line with previous findings in the literature, e.g. Huang et al. [27] that document that withholding ‘biasing’ information improves impartial decision making. In addition, our finding that a larger weight for distributional considerations is obtained when participants choose for a close relative or friend as compared to when they choose for themselves supports the idea that people making high risk decisions for others are less risk-seeking than those who make similar decisions for themselves [28], particularly when decisions are on health-related matters [29].

Previous studies in the health domain, e.g. [9] and [16], document limited or no positional concerns in health-related decisions. The main argument provided for limited positional concern in the health domain is that healthcare is considered as a basic service and positional concerns are more likely to be exhibited when one already possesses a subsistence or sufficient level of a good or service. In contrast, we found a significant degree of positional concerns among participants in our choice experiment. While additional research is needed to confirm this result, we note that the way in which we conceptualized positional concerns in our study slightly differs from previous literature: we considered both the disutility from a disadvantageous inequality and the utility from an advantageous inequality instead of the common approach of defining positional behaviour just as the utility from having ‘more’ than others. The conceptualization of positional concerns adopted in this study is important for understanding redistribution effects of a policy when decision makers evaluate disadvantageous and advantageous inequalities of equal magnitude asymmetrically. For instance, a Pareto-improving unequal distribution policy can be welfare-reducing if the disutility from a disadvantageous inequality looms larger than the utility from an advantageous inequality of equal magnitude. While we did not find evidence of such asymmetric weighing in our study, this remains an interesting avenue for further work.

Finally, the comparison between the respective weights for self-interest, positional concerns and distributional considerations of the US and the UK sample revealed that UK respondents place relatively larger weight on self-interest (in the socially inclusive personal perspective) and distributional considerations (in both socially inclusive personal and social perspectives). This result could reflect differences in societal values underlying the healthcare systems in the UK and the US; however, a full interpretation of this finding in light of differences between the countries’ societal values and healthcare systems, as suggested by Hofstede [21], is outside the scope of this paper. One could, however, argue that the finding that self-interest as well as distributional considerations matter in both countries signals that a mix of private initiative and government intervention in the financing and organization of healthcare services is generally preferred over strictly privately or publicly funded healthcare systems, with the actual mix being associated with the local importance of societal values like solidarity and independence. Obviously, this argument needs to be tested further, and it is also important to acknowledge that the relative importance of the three motivations may differ, for example, across the contexts of communicable and non-communicable diseases. For example, the burden of the recent pandemic on healthcare systems across the world was an extraordinary situation, but decisions by governments about measures to mitigating the spread of the virus and allocating scarce healthcare resources differed considerably between countries [30].

To sum up, this study recognized and disentangled the role of overlapping individual and collective interests as well as positional concerns in decision making in the healthcare context and offers a methodological (data collection and analysis) approach to identify the relative importance of these three motivations. We used the approach to empirically assess individual preferences related to access to healthcare services and presented the results of our investigation. However, given that our empirical exposition is based on a hypothetical scenario, we re-iterate that readers should exercise caution in interpreting our results. Most importantly, future research based on a similar design in the health domain as well as in other domains is essential to establish our results and to draw meaningful policy recommendations.

## Data Availability

The data that support the findings of this study are available from the corrosponding author upon request.

## A1

Estimated parameters and standard errors for different number of artificial decision makers (i.e. simulated choices) (Figs. 4, 5).

## A2

### Design B choice tasks for Group 1

We now ask you to answer a series of 15 choice questions as before, but this time you will have information about your specific position in the distribution, that is, your own waiting time if you would be affected by the disease. Importantly, we do not ask you to assume that you are affected by the disease: you are merely part of the group that may become affected. Your own waiting time would be specified (in red) as follows. For clarity, we give you the following choice example [all scenarios have single option].

In the example, plan A indicates that 70% of people affected by the disease would have to wait 3 weeks for treatment, 5% 6 weeks, 10% 9 weeks, 5% 12 weeks and the remaining 10% 15 weeks. If you are among the affected, you would have to wait 6 weeks to receive treatment. Plan B means that 5% of people affected by the disease would have to wait 3 weeks for treatment, 5% 6 weeks, 5% 9 weeks, 80% 12 weeks and the remaining 5% 15 weeks. If you are among the affected, you would have to wait 9 weeks to receive treatment. The question to you would be, which plan do you prefer to be implemented?

Next, we ask you to make such a choice for 15 similar set of two plans.

#### Scenario 1

Which plan do you prefer?

#### Scenario 2

Which plan do you prefer?

#### Scenario 3

Which plan do you prefer?

#### Scenario 4

Which plan do you prefer?

#### Scenario 5

Which plan do you prefer?

#### Scenario 6

Which plan do you prefer?

#### Scenario 7

Which plan do you prefer?

#### Scenario 8

Which plan do you prefer?

#### Scenario 9

Which plan do you prefer?

#### Scenario 10

Which plan do you prefer?

#### Scenario 11

Which plan do you prefer?

#### Scenario 12

Which plan do you prefer?

#### Scenario 13

Which plan do you prefer?

#### Scenario 14

Which plan do you prefer?

#### Scenario 15

Which plan do you prefer?

## A3

See Table 6.

**Table 6:** Descriptive statistics on sample characteristics

Variable		Group-1 (*N* = 1552)		Group-2 (*N* = 1540)	
		US-1 (*N* = 769)	UK-1 (*N* = 783)	US-2 (*N* = 773)	UK-2 (*N* = 767)
Gender	Male	50.5%	49.4%	45.1%	52.3%
	Female	49.5%	50.5%	54.9%	47.7%
	Other	0%	0.1%	0%	0%
Age	Average	40 years	40.1 years	45.2 years	46.4 years
Education	Bachelor’s and above	54.5%	51.5%	39.7%	40.9%
Marital status	Single	30.2%	32.8%	33.9%	29.3%
	Married	60.2%	61.9%	52%	62.2%
	Divorced	8.1%	4.5%	11%	7.2%
	Widowed	1.5%	0.8%	3.1%	1.3%
Household with children		58.4%	58.1%	37.9%	41.1%
Employment condition*	Permanent	77%	85.8%	41.7%	61%
	Temporary	6.5%	5.5%	6.6%	3.9%
	Self-employed	16.5%	8.7%	11%	5.9%
Ability to make ends meet with income	Very difficult	19%	13%	16.3%	12%
	Slightly difficult	35.2%	33.1%	36.9%	34.9%
	Fairly easy	27.7%	35.9%	30.4%	37.2%
	Easy	18.1%	18%	16.4%	15.9%
Health insurance	Private	67.1%	43.7%	48.4%	33.4%
	Medicare	11.3%	–	26.9%	–
	Medicaid	13.9%	–	16%	–
	None	7.7%	56.3%	8.7%	66.6%

## A4

### Formulas used to calculate distributional metrics

Let $m=1, 2, \dots .., M$ represent the number of levels of waiting times and ${T}_{\mathrm{mit}}$ denote the waiting time corresponding to $m$ in alternative $i$ of choice task $t$. Suppose that the share of patients having a waiting time of ${T}_{\mathrm{mit}}$ is ${\omega }_{\mathrm{mit}}$.

The expected value ($E$) of waiting time distribution represented by alternative $i$ in choice task $t$ is computed by simply taking the weighted average of the waiting times.

$${E}_{it}=\sum_{m=1}^{M}{\omega }_{\mathrm{mit}}{T}_{\mathrm{mit}}.$$

For example, the expected waiting times for the example choice task is computed as.

Plan A: $$0.7*3+0.05*6+0.1*9+0.05*12+0.1*15=5.4 \mathrm{weeks}.$$

Plan B: $$0.05*3+0.05*6+0.05*9+0.8*12+0.05*15=11.25 \mathrm{weeks}.$$

Given the information above the **Sh**annon entropy (*H*) value for waiting time distribution represented by alternative $i$ in choice task $t$ is given by the following formula [31]:

$${H}_{it}=-\sum_{m=1}^{M}{\omega }_{\mathrm{mit}}\mathrm{log}({\omega }_{\mathrm{mit}}).$$

The entropy for the example choice task, for example, is

Plan A: $$-(0.7*\mathrm{log}\left(0.7\right)+0.05*\mathrm{log}\left(0.05\right)+0.1*\mathrm{log}\left(0.1\right)+0.05*\mathrm{log}\left(0.05\right)+0.1*\mathrm{log}\left(0.1\right))=1.01.$$

Plan B: $$-(0.05*\mathrm{log}\left(0.05\right)+0.05*\mathrm{log}\left(0.05\right)+0.05*\mathrm{log}\left(0.05\right)+0.8*\mathrm{log}(0.8)+0.05*\mathrm{log}\left(0.05\right))=0.778.$$

The generalized entropy (GE) inequality index corresponding to different values of the sensitivity parameter ($\alpha$) is computed using the following formulas:

$${\mathrm{GE}}_{it}(\alpha ) =\left\{\begin{array}{c}\frac{1}{\alpha \left(\alpha -1\right)}{\sum }_{m=1}^{M}{\omega }_{\mathrm{mit}}\left[{\left(\frac{{T}_{\mathrm{mit}}}{{E}_{it}}\right)}^{\alpha }-1\right],\alpha \ne 0, 1\\ {\sum }_{m=1}^{M}{\omega }_{\mathrm{mit}}\left(\frac{{T}_{\mathit{mit}}}{{E}_{\mathit{it}}}\right)\mathrm{ln}\left(\frac{{T}_{\mathrm{mit}}}{{E}_{it}}\right),\alpha =1\\ -{\sum }_{m=1}^{M}{\omega }_{\mathrm{mit}}\mathrm{ln}\left(\frac{{T}_{\mathrm{mit}}}{{E}_{it}}\right),\alpha =0,\end{array}.\right.$$

The parameter $\alpha$ regulates the sensitivity of the index to levels of waiting times. For a positive and large (small) $\alpha$, the $GE$ index is sensitive to changes in the upper (lower) tail of the distribution. Note that $GE$ ranges between 0 and $\infty$, and a larger value of $\mathrm{GE}$ indicates larger inequality in a distribution.

For the example choice task and $\alpha =10$, GE inequality index corresponding to Plan A and B is calculated as follows.

Plan A: $$\frac{1}{10*\left(10-1\right)}\left(0.7*\left({\left(\frac{3}{5.4}\right)}^{10}-1\right)+0.05*\left({\left(\frac{6}{5.4}\right)}^{10}-1\right)+0.1*\left({\left(\frac{9}{5.4}\right)}^{10}-1\right)+0.05*\left({\left(\frac{12}{5.4}\right)}^{10}-1\right)+0.1*\left({\left(\frac{15}{5.4}\right)}^{10}-1\right)\right)=32.2.$$

Plan B: $$\frac{1}{10*\left(10-1\right)}\left(0.05*\left({\left(\frac{3}{11.25}\right)}^{10}-1\right)+0.05*\left({\left(\frac{6}{11.25}\right)}^{10}-1\right)+0.05*\left({\left(\frac{9}{11.25}\right)}^{10}-1\right)+0.8*\left({\left(\frac{12}{11.25}\right)}^{10}-1\right)+0.05*\left({\left(\frac{15}{11.25}\right)}^{10}-1\right)\right)=0.016.$$

## A5

See Table 7.

**Table 7:** Test of asymmetry between advantageous and disadvantageous equal magnitude inequality

Group	Parameter	$\mu =0.01$		$\mu =0.1$		$\mu =0.5$		$\mu =1$		$\mu =2$		$\mu =5$	
		US	UK	US	UK	US	UK	US	UK	US	UK	US	UK
		Estimate (*t* ratio)	Estimate (*t* ratio)	Estimate (*t* ratio)	Estimate (*t* ratio)	Estimate (*t* ratio)	Estimate (*t* ratio)	Estimate (*t* ratio)	Estimate (*t* ratio)	Estimate (*t* ratio)	Estimate (*t* ratio)	Estimate (*t* ratio)	Estimate (*t* ratio)
One	$\beta$	− 0.028 (− 2.3)	− 0.011 (− 1.8)	− 0.047 (− 5.9)	− 0.039 (− 5.1)	− 0.056 (− 7.9)	− 0.043 (− 6.4)	− 0.055 (− 8.4)	− 0.042 (− 6.7)	− 0.054 (− 8.7)	− 0.042 (− 6.8)	− 0.054 (− 8.8)	− 0.042 (− 6.9)
	$\mu$	Fixed	Fixed	Fixed	Fixed	Fixed	Fixed	Fixed	Fixed	Fixed	Fixed	Fixed	Fixed
	Final log-likelihood	− 7988.6	− 8137.0	− 7968.0	− 8122.3	− 7957.2	− 8117.2	− 7956.2	− 8116.7	− 7955.8	− 8116.6	− 7955.6	− 8116.5
	Observations	11,535	11,745	11,535	11,745	11,535	11,745	11,535	11,745	11,535	11,745	11,535	11,745
Two	$\beta$	− 0.025 (− 2.1)	− 0.009 (− 1.8)	− 0.048 (− 5.9)	− 0.037 (− 4.8)	− 0.056 (− 7.9)	− 0.039 (− 5.8)	− 0.055 (− 8.4)	− 0.039 (− 6.1)	− 0.054 (− 8.7)	− 0.038 (− 6.3)	− 0.054 (− 8.8)	0.038 (− 6.3)
	$\mu$	Fixed	Fixed	Fixed	Fixed	Fixed	Fixed	Fixed	Fixed	Fixed	Fixed	Fixed	Fixed
	Final log-likelihood	− 8030.4	− 7971.2	− 8009.4	− 7958.7	− 7998.8	− 7955.1	− 7997.8	− 7954.8	− 7997.4	− 7954.7	− 7997.2	− 7954.7
	Observations	11,595	11,505	11,595	11,505	11,595	11,505	11,595	11,505	11,595	11,505	11,595	11,505

## A6

See Table 8.

**Table 8:** Results when controlling for between-country and between-group potential scale differences (SI + PC + DC (expected value))

Parameters and final log likelihood (LL)	Groups								Pooled (group one + group two)		Pooled with scale (group one + group two)	
	Group one				Group two							
	US	UK	Pooled (US + UK)	Pooled with scale (US + UK)	US	UK	Pooled (US + UK)	Pooled with scale (US + UK)	US	UK	US	UK
	Est (*t* ratio)	Est (*t* ratio)	Est (*t* ratio)	Est (*t* ratio)	Est (*t* ratio)	Est (*t* ratio)	Est (*t* ratio)	Est (*t* ratio)	Est (*t* ratio)	Est (*t* ratio)	Est (*t* ratio)	Est (*t* ratio)
${\beta }^{SI}$	0.194 (8.3)	0.335 (14.1)	0.263 (15.7)	0.194 (12.9)	0.326 (13.8)	0.323 (13.4)	0.325 (19.1)	0.291 (16.1)	0.259 (15.5)	0.329 (19.3)	0.208 (13.3)	0.314 (17.1)
${\beta }^{PC}$	0.193 (7.4)	0.148 (5.4)	0.170 (9.1)	0.115 (7.4)	0.181 (6.7)	0.194 (7.0)	0.187 (9.6)	0.169 (9.2)	0.186 (9.9)	0.170 (8.7)	0.142 (8.5)	0.164 (8.6)
${\beta }^{DC}$	0.267 (11.2)	0.487 (19.8)	0.376 (22)	0.281 (16)	0.408 (16.6)	0.568 (22.4)	0.487 (27.6)	0.444 (23.1)	0.336 (19.7)	0.527 (29.9)	0.269 (15.2)	0.505 (24.8)
Scale (UK)				0.537(8.3)				0.191(3.9)				
Scale (Group two)											0.418 (6.5)	0.087 (1.9)
Final log likelihood	− 7803.8	− 7632.4	− 15,490.8	− 15,450	− 7619.3	− 7392.0	-15,026.5	-15,018.5	− 15,450.9	− 15,027.1	− 15,427.7	− 15,025.3
Observations	11,535	11,745	23,280	23,280	11,595	11,505	23,100	23,100	23,130	23,250	23,130	23,250
^‡^Swait and Louviere test	${H}_{1A}$: 27.7*** Preference structures differ after controlling for scale differences				${H}_{1A}$: 14.4*** Preference structures differ after controlling for scale differences						H_1A_: 9.2* Preference structures differ after controlling for scale	H_1A_: − 1.8 H_1B_: 3.6* Marginal difference in only scale

^‡^The null hypothesis for this test, ${H}_{1}: {\beta }_{\mathrm{US}}={\beta }_{\mathrm{UK} }\& {\mu }_{\mathrm{US}}={\mu }_{\mathrm{UK}}$, $\mu$ denoting scale can be tested in two stages. ${H}_{1A}: {\beta }_{\mathrm{US}}={\beta }_{\mathrm{UK}}$ tests for similar preference structure after controlling for scale differences. If ${H}_{1A}$ is rejected, ${H}_{1}$ will also be rejected. If ${H}_{1A}$ cannot be rejected, ${H}_{1B}: {\mu }_{\mathrm{US}}={\mu }_{\mathrm{UK}}$ tests for scale homogeneity. The same holds for between groups

## Footnotes

1 i.e. the choice is between a state of the world where a decision maker is better off in absolute outcomes than others and a state of the world where the decision maker is better off in relative terms, but worse off in absolute outcomes than the first state.

2 The EuroQol EQ-5D-5L instrument consists of five dimensions of health status, namely, mobility, self-care, usual activities, pain/discomfort and anxiety/depression. For each dimension, there are five different levels: no problems, slight problems, moderate problems, severe problems and extreme problems.

3 For example, we show that the model parameters used for simulation can be efficiently estimated with 3,000 simulated choices (i.e. 200 decision makers).

4 Before fully deploying the survey, we analysed data collected from about 10% of respondents for each sub-sample to check that the scenarios were understood properly as well as to make adjustments if necessary; we did not find any issues.
